# DNA Nanostructures for siRNA Delivery

**DOI:** 10.1021/acs.bioconjchem.6c00044

**Published:** 2026-04-08

**Authors:** Bharath Raj Madhanagopal, Sarah Youssef, Arun Richard Chandrasekaran

**Affiliations:** † Department of Nanoscale Science and Engineering, University at Albany, State University of New York, Albany, New York 12222, United States; ‡ The RNA Institute, University at Albany, State University of New York, Albany, New York 12222, United States

## Abstract

Small interfering RNAs (siRNAs) represent an emerging class of versatile nucleic acid drugs for a broad spectrum of genetic and metabolic disorders. Since siRNAs can be developed to silence any target gene with relative ease compared to conventional drugs, there is enormous potential in this therapeutic modality for combating a variety of illnesses. However, its application is limited by low biostability, rapid clearance, and poor biodistribution of naked RNA. This is overcome by employing backbone modifications, conjugation of cell-targeting ligands, and the use of nanocarriers. DNA-based nanostructures are well suited to carry siRNA drugs since the use of DNA as a construction material provides the ability to tune the size, shape, and other morphological features of the nanostructure. DNA nanostructures also allow easy loading of multiple siRNA drugs with stoichiometric precision, enable functionalization with various targeting and tracking agents, and can be designed to deliver siRNA cargo in response to various stimuli. In this review, we provide an overview of recent reports on the use of DNA-based nanostructures to achieve targeted delivery of siRNA in vitro and in vivo. We discuss aspects of nanostructure design for various drug-loading and drug-release strategies and pharmacodynamic and pharmacokinetic properties of DNA nanocarriers and provide a survey of various diseases that have been targeted by siRNA-carrying DNA nanostructures. We also highlight the challenges facing these new-generation nanocarriers in achieving their therapeutic potential and clinical applications.

## Graphical abstract

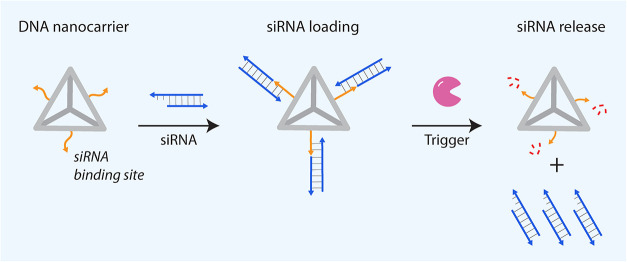

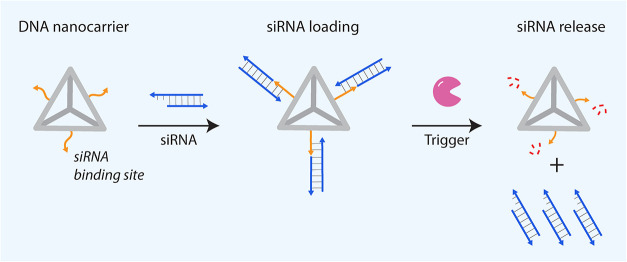

## Introduction

The balance between health and disease often hinges on the function and regulation of one or more genes. For diseases caused by aberrant gene regulation or dysfunction, a therapeutic strategy that specifically modulates the affected genes allows precise control over the disease progression and clinical outcome. Such direct interventions into the molecular processes underlying a disease are enabled by nucleic acid therapeutics.^1,2^ In this rapidly growing category of modern medicine, RNA interference (RNAi) through small interfering RNA (siRNA)^3–5^ has emerged as one of the most promising therapeutic modalities for a variety of genetic and metabolic disorders, such as hypercholesterolemia, primary hyperoxaluria type 1, acute hepatic porphyria, and hereditary transthyretin amyloidosis.^6^

Much like the unerring arrows of Legolas in Tolkien’s epic The Lord of the Rings, siRNAs strike their intended target mRNA with remarkable precision and achieve sequence-specific gene silencing.^7,8^ Gene silencing by RNAi is achieved by the delivery of synthetic siRNAs that are 21–23-bp-long double-stranded RNA molecules and involves four major steps: formation of the RNA-induced silencing complex (RISC), activation of the RISC, target recognition, and target cleavage (Figure 1). siRNAs comprise a sense (passenger) strand that has the same sequence as the target RNA and a complementary antisense (guide) strand.^7,9^ Inside the target cells, siRNA binds to the Argonaute 2 (AGO2) protein to form RISC. During RISC assembly, the sense strand (passenger strand) of the siRNA is cleaved, and the antisense strand guides RISC to the complementary mRNA by sequence recognition. The catalytic action of AGO2 within RISC cleaves the target mRNA to achieve sequence-specific gene knockdown. Because siRNA molecules remain stable for weeks as RISC and target multiple transcripts, a sustained knockdown of the target gene can be achieved by only a few hundred siRNAs per cell.^10^ Since the targeting mechanism of siRNAs is complementary base pairing, they can be used to target any gene, and the therapeutic potential of this modality is therefore vastly greater than that of conventional small-molecule drugs. Further, siRNA activity involves enzymes endogenous to cells and thus does not require the delivery of large enzymes, and since siRNA interferes with mature mRNA, it requires only cytoplasmic delivery, which is easier to achieve than nuclear delivery.

**Figure 1:**
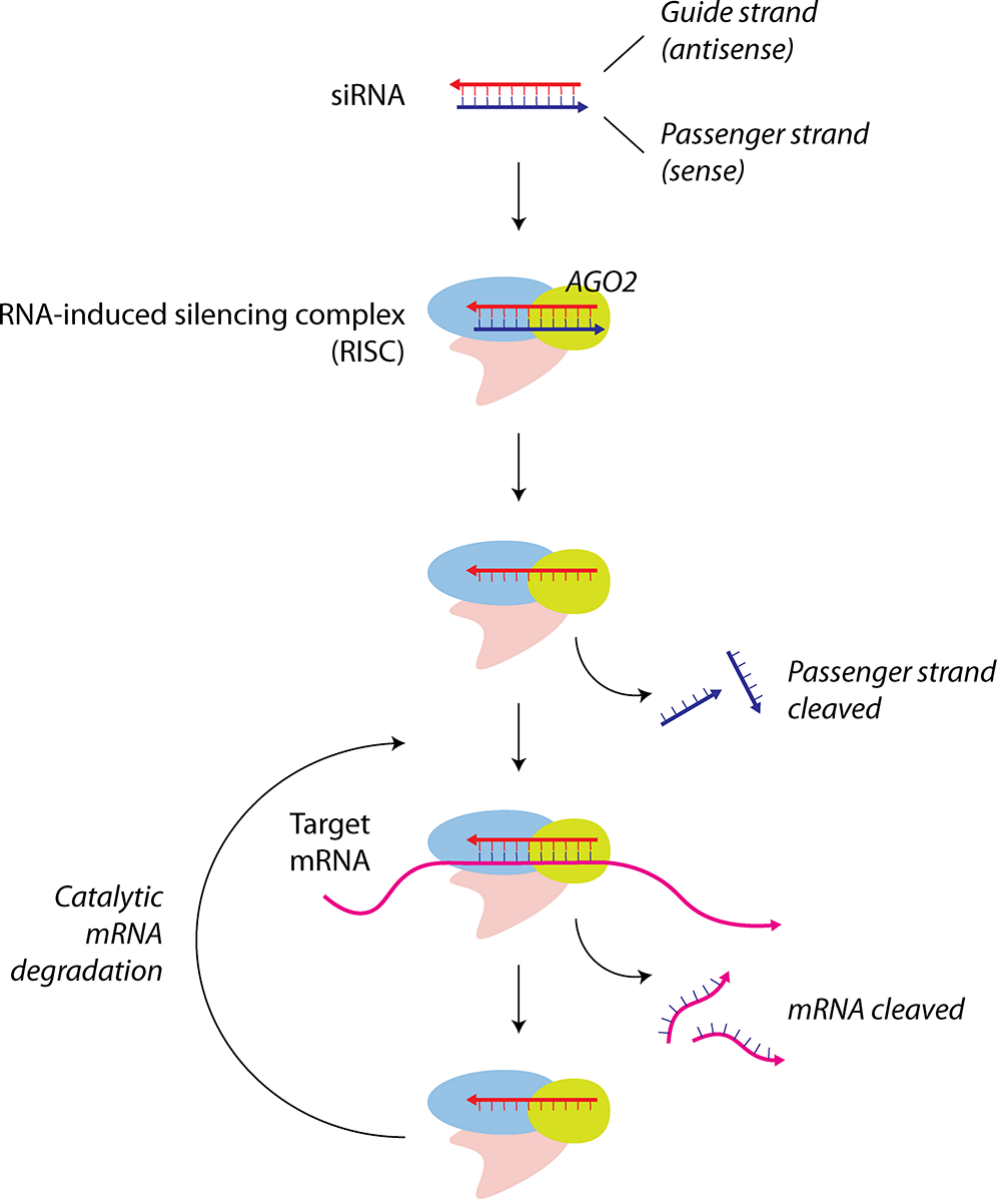
Overview of siRNA-mediated gene silencing by RNA interference (RNAi).

While siRNAs have emerged as promising therapeutics for genetic disorders, cancers, and infectious diseases over the past couple of decades, major hurdles to using siRNAs as drugs are their short half-life in serum (∼15 min) and rapid clearance by the kidneys.^11^ Further, their relatively large molecular weights (∼12 kDa) and high negative charge make delivery across the plasma membrane and escape from endosomes challenging, contributing to their poor biodistribution and bioavailability. Although short double-stranded RNAs are not as immunogenic as longer RNA molecules,^12^ the immunogenicity of unmodified siRNAs can pose a challenge for using them as therapeutics. Despite their high specificity, siRNAs may exhibit off-target effects through sequence-dependent and sequence-independent interactions. Careful design considerations include the elimination of cross-hybridization of the antisense strand of the siRNA to nontarget mRNAs and the binding of siRNAs to cellular proteins.^13–15^ These challenges are overcome typically by two means: (1) incorporating chemical modifications into the backbone of siRNAs to improve thermal stability, biostability, pharmacokinetics, pharmacodynamics, and target specificity; (2) using a nanocarrier that protects the therapeutic siRNAs and delivers them to the target cells.

Improving the pharmacokinetics and cellular uptake properties of siRNAs is crucial for realizing the therapeutic potential of siRNAs. Chemical modifications to the sugar–phosphate backbone of RNA, including 2′O-methyl ribose, locked nucleic acid, and phosphorothioate, enhance the therapeutic properties of siRNA drugs and are routinely used.^11,16^ Targeting siRNAs to specific tissues has been another major obstacle that limits their therapeutic potential. Terminal functionalization of the sense or antisense strands with targeting moieties, such as *N*-Acetyl galactosamine (GalNAc), aptamers, antibodies, and lipid groups, influences the biodistribution of siRNAs and increases their accumulation in specific tissues.^16^ Despite the above-mentioned strategies, the need to achieve controlled siRNA stoichiometry and carry additional elements such as targeting and cell-penetrating agents would require a multifunctional delivery carrier. The use of nanocarriers also obviates the need for functionalization of the therapeutic siRNA itself, and it can be used to guide the delivery of the siRNAs to the desired targets. An ideal nanocarrier must be biocompatible, nontoxic, and nonimmunogenic, and should bypass rapid hepatic and renal clearance while accumulating specifically in the target tissue and facilitating the delivery of siRNA cargo to the cytosol of the target cells.

Effective siRNA delivery has been accomplished through viral vectors,^17^ but has several drawbacks including complexity of vector preparation, safety concerns, and the generation of immune and inflammatory responses when used in vivo.^18^ Existing nonviral delivery systems such as liposomes,^19^ dendrimers,^20^ polymers,^21^ and nanoparticles^22^ have drawbacks such as toxicity, inefficient drug loading, and immunogenicity.^23^ There is a need for nonviral RNAi carriers that can protect the siRNA and facilitate the delivery and uptake of the siRNA into target cells.^24,25^ Recently, DNA nanostructures have emerged as a promising class of functional materials with tunable drug delivery properties.

## DNA Nanocarriers

DNA nanostructures are promising multifunctional drug carriers that are typically constructed by assembling multiple DNA strands guided by their sequence.^26^ The complementary base-pairing properties and the well-studied double helical structure of DNA make it an ideal material to build highly complex nanoscale objects with excellent control over the size and morphology.^27^ Based on the design considerations and structural complexity, DNA nanocarriers can be of many types, including polyhedral DNA nanostructures, tiles, DNA origami, spherical nucleic acids, nanogels, and nanostrings. These DNA nanostructures can be created through different strategies.^28^ In cooperative assembly, multiple oligonucleotides hybridize to yield the nanostructure (Figure 2a).^29^ In modular self-assembly, multiple subcomponents are created first, followed by their assembly into the target DNA nanostructure (Figure 2b).^30^ For larger arrays and structures, small DNA motifs can be connected to each other to form higher-order DNA structures via hierarchical self-assembly (Figure 2c).^31^ In the now common DNA origami strategy, a long single-stranded scaffold is folded into arbitrary shapes by short complementary staple strands (Figure 2d).^32^ Another assembly strategy uses single-stranded DNA bricks that can self-assemble into finite objects and infinite arrays (Figure 2e).^33^

**Figure 2:**
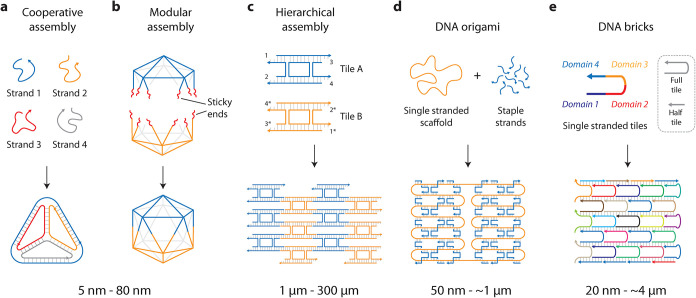
DNA nanostructures. (a) DNA nanostructures assembled by cooperative assembly of short DNA single strands (e.g., a tetrahedron). (b) Modular assembly of DNA motifs into polyhedral structures (e.g., an icosahedron). (c) Hierarchical assembly of DNA motifs into 2D arrays. (d) Folding of a long single-stranded DNA scaffold into arbitrary shapes using the DNA origami strategy. (e) Single-stranded DNA built into larger structures using the DNA brick strategy.

Easy chemical synthesis, the ability to incorporate chemical modifications, the availability of enzymes to manipulate the structure, and a good understanding of the supramolecular assembly behavior contribute to the growth of DNA nanostructures into versatile nanocarriers. Compared with other nanomaterials, DNA-based nanostructures are easier to design. While the programmable assembly of DNA strands into 1D, 2D, and 3D structures is still being perfected, the morphological and topological control achieved with DNA is unparalleled compared to that of other materials used for drug delivery. Because the DNA double helix is only 2 nm wide, nanostructures can be easily constructed using DNA with nanoscale precision. Recent advances in the field of nucleic acid chemistry have enabled the synthesis of chemically modified DNA and RNA molecules that show high nuclease resistance without compromising their thermal stability, a key parameter for biological applications. By incorporating chemical modifications into nanostructures^34^ and utilizing DNA analogues^35^ to construct them, nanocarriers with tunable biostability can be synthesized, offering controlled drug delivery properties.

Tetrahedral DNA nanostructures are among the most widely used polyhedral nanostructures for drug delivery applications.^36^ They are typically made with four strands of DNA and can vary in size between 10 and 50 nm.^37^ The compact nature of DNA tetrahedra impairs nuclease degradation and improves its biostability.^38,39^ Compared to other structures such as cubes, icosahedra, and buckyballs, tetrahedra are taken up by cells more effectively, making them a preferred DNA nanostructure for drug delivery applications.^40^ DNA tetrahedra also exhibit favorable immunogenic properties, low cytotoxicity, and anti-inflammatory properties.^41,42^ Larger DNA origami structures allow the loading of a large number of cargo duplexes and other functionalities with the ability to control nanostructure morphology and spatial resolution between attached functional groups.^43^ In other examples, DNA-polymer conjugates and nanogels made from DNA-grafted polymer brushes have also been used as nanocarriers.^44^

## Pharmacokinetics and Pharmacodynamics of DNA Nanostructures

Pharmacokinetics describes how biological systems interact with DNA nanostructures and generally includes administration, distribution, metabolism, and elimination. While DNA nanostructures have been explored for biomedical applications, a thorough analysis of the pharmacokinetics of DNA nanostructures is lacking.^45^ Key studies have analyzed the effects on the biostability, therapeutic efficiency, cytotoxicity, and immunogenic responses of DNA nanostructures (Figure 3). In these studies, the structural design parameters of DNA nanostructures, such as size, shape, surface chemistry, and appended functionalities, have played a significant role in determining the pharmacokinetic behavior of DNA nanostructures. Specific to the pharmacokinetics of siRNA delivery systems, in addition to biodistribution and clearance, the main determinants of successful siRNA therapeutics entail biostability until reaching target sites, efficient cell uptake and endosomal escape to cytoplasm, release from carriers inside cytoplasm, biocompatibility, and low immunogenicity.

**Figure 3:**
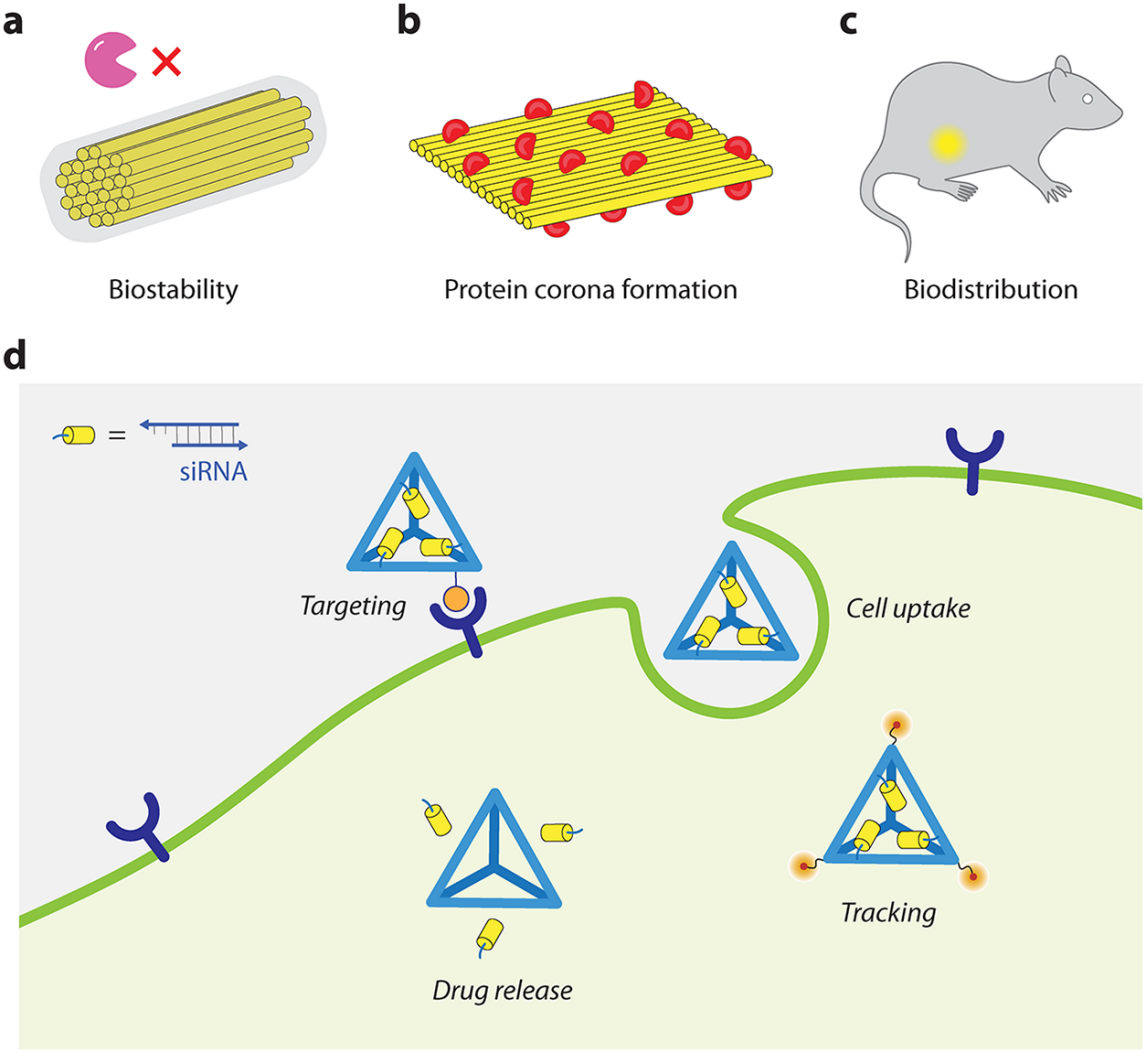
Overview of drug delivery pathway using DNA nanostructures. DNA nanostructures can be functionalized to be (a) biostable against nuclease activity, (b) survive protein corona formation, and (c) used as imaging modalities. (d) DNA nanostructures with siRNA cargos can be targeted to specific cells for uptake, tracking, and disassembly or degradation in response to external stimuli to release the cargo.

### Biostability

Stability of DNA nanocarriers in the host is one of the most important challenges for efficient drug delivery. DNA nanostructures show higher nuclease resistance compared to double-stranded DNA and can last for several hours, making them suitable for siRNA delivery.^46^ For example, DNA tetrahedra with 20-bp-long edges showed nearly fifty-fold higher stability compared to double-stranded DNA control in 10% FBS.^47^ siRNA encapsulated within a DNA tetrahedra nanocarrier remained intact for 1 h in the presence of RNase A, while the nanocarrier itself was intact for 6 h in 10% FBS.^48^ Further, DNase I-mediated degradation can be avoided by constructing the nanostructures using artificial nucleic acid (XNA) backbones such as l-DNA,^49^ threoninol nucleic acid (TNA),^50^ and peptide nucleic acid (PNA).^51^ While nanostructures have been constructed using these XNAs, there are only a few reports of their use as nanocarriers for siRNA delivery. The biostability of DNA nanocarriers is also influenced by the small-molecule drugs that they carry. DNA nanotubes synthesized in the presence of metformin showed higher biostability, remaining intact in 10% FBS for up to 24 h and accumulating at the tumor sites within 1 h of administration.^52^ Similarly, the addition of minor groove binders improved the stability of wireframe DNA origami structures.^53^ Additionally, the counterions used for assembling DNA and DNA-XNA nanostructures can affect their stability against nucleases. DNA assembled using monovalent Na^+^ as counterions or in a hydrated ionic liquid resisted degradation by DNase I.^54,55^ The size of DNA nanostructures can also influence biostability, with DNA tetrahedra containing shorter edges being more biostable compared to structures with longer edges.^39^ Moreover, the biostability of DNA nanostructures can be tuned by the design of the structural motif. The placement of crossovers between two adjacent double helices at every half-turn in a paranemic crossover architecture leads to enhanced stability against nucleases.^56^ Another example is switchback DNA, a left-handed DNA double helix with half-turns linked laterally. Switchback DNA shows a slightly higher stability against DNase I compared to double-stranded DNA.^57^ These structural properties can be used to improve the design of DNA nanocarriers by integrating these motifs in the nanostructure design. In addition to these strategies, DNA nanostructure stability can be enhanced by chemical ligation to ligate nick points^47^ or by coating with lipids,^58^ proteins,^59,60^ or polymers such as poly­(l-Lysine) and poly­(ethylene glycol)-polylysine.^61–63^

### Protein Corona Formation

Upon DNA nanostructure administration, the structures are prone to plasma protein binding, resulting in the protein corona formation. Initially, a rapid soft protein corona is formed that is later replaced by a hard, more stable corona.^64^ The type of protein corona determines the circulation time and, consequently, the therapeutic efficiency of the administered DNA nanostructures. For instance, among the common serum proteins termed opsonins are immunoglobulins that recruit immune cells such as phagocytes, eventually leading to rapid immunological clearance before nanoparticles can reach their intended site of action. The types of proteins involved would primarily depend on the surface charge of nanoparticles and hence affect the pharmacokinetic behavior.^64^ In case of DNA nanostructures, more cationic proteins are recruited in the corona due to the negative charge of DNA.^65^

To rationally develop better DNA nanostructures for drug delivery, a better understanding of the factors responsible for protein corona formation is required. Comparison of DNA tetrahedra, flat origami sheet, compact and hollow 3D origami rods indicated that the size and shape of DNA nanostructures did not significantly affect the composition of protein corona formation around the nanostructures.^65^ However, the surface charge of the nanostructure seemed to play a key role in protein corona formation, and this character can be modulated to be hydrophobic (by attaching cholesterol moieties) or cationic/hydrophilic (by addition of lysine-PEG). For example, a DNA tetrahedron functionalized with three cholesterol molecules resulted in the formation of a lipoprotein-rich corona and showed preferential liver accumulation.^66^ Similarly, the addition of lysine-PEG resulted in the recruitment of smaller proteins due to the water sheath layer created by PEG that shields the nanostructures against larger proteins.^67^ However, both unmodified and modified DNA nanostructures showed universal adsorption of immunoglobulins responsible for opsonization and rapid immunological clearance. Specific protein composition can also influence macrophage uptake, and predesigned protein corona (e.g., clusterin corona) can significantly influence opsonization and cell uptake.^67,68^ Thus, modulating the DNA surface can affect the type of protein corona formed, relative tissue distribution, and cell uptake.

### Cell Uptake

Several factors affect cellular uptake of DNA nanostructures, with the size and shape of the nanostructure playing a significant role. For example, DNA six-helix bundles showed the highest uptake compared to a 3-point star motif and a DNA tetrahedron across different cell types.^69^ Further, large DNA origami structures such as rods and tripods were taken up more efficiently compared to smaller multistranded DNA bundles and tetrahedra.^70^ The placement of siRNA cargo on the DNA nanostructure can also influence the uptake efficiency. Incorporation of the siRNA on the edges of a tetrahedral nanocarrier was more favorable compared to attachments to the outer surface.^48^ In addition to these factors, cellular uptake of DNA nanostructures can also be enhanced by targeted delivery using antibodies, aptamers or cell-penetrating peptides.^63,71,72^

### Endosomal Escape

In order to perform intracellular functions, siRNA within DNA nanostructures are required to escape endosomes and get released inside the cytoplasm.^73^ However, most DNA nanostructures are taken up through scavenger receptors via endocytosis and eventually end up degrading inside lysosomes, the end stage of endosomal uptake. Endosomes trap ∼99% of therapeutic RNA and constitute a major structural barrier for all charged nanocarriers as well.^74^ Conjugation of cationic peptides and introducing phosphorothioate modifications in the backbone are some of the strategies used to enhance endosomal escape of macromolecules.^75,76^ Because DNA nanocarriers can be easily functionalized with various moieties and chemically modified at defined sites, the endosomal barrier can be tackled by surface engineering of the nanostructures to incorporate these features. Coating DNA nanostructures with cationic polymers such as polyethylenimine enables their escape from the endosomes.^77,78^ In contrast, l-DNA tetrahedra were able to escape the endosomes without any coating or endosmolytic agent although the mechanism is not clear.^49^ Typically, DNA tetrahedra are taken up by caveolin-mediated endocytosis,^79^ but when synthesized in the presence of spermidine, the tetrahedra were taken up through clathrin-mediated endocytosis.^80^ In addition, endosmolytic agents such as aurein,^81^ cationic lipids^82^ and histidine-grafted polymers^77^ can be used to achieve cytosolic delivery by lysis of the endosomal membrane.^83^

An alternative pathway is through direct cytosolic uptake. When coated with bioreducible polymers such as poly­(cystaminebis­(acrylamide)-1,6-diaminohexane), ∼40% of internalized DNA nanostructures were present in the cytoplasm rather than in vesicular compartments.^63^ Similarly, disulfide-containing units can be added to DNA tubes, promoting direct cytosolic delivery.^84^ The disulfide bonds allow for exchange with thiols on cell membrane proteins leading to direct internalization of DNA nanostructures inside the cell cytoplasm bypassing endocytic pathway.^85^ Addition of membrane-anchoring moieties such as cholesterol or ligands binding cell-surface glycocalyx also increased the internalization efficiency and intranuclear delivery.^86^ Further, a DNA origami needle that mimics bacteriophages was used to release the cargo nucleic acid molecules directly inside the cytoplasm bypassing endocytosis.^87^

### Immunogenicity

The immunogenicity of DNA nanostructures is dependent on several factors, including shape and dosage, with some studies indicating minimal innate immune stimulation,^88^ while others imply that DNA nanostructures can trigger an immune response.^89^ One reason behind the minimal immune response against DNA nanostructures is that the compactness of these designs, compared to double-stranded DNA, shows lower interactions with Toll-like receptor 9 (TLR9), which is responsible for foreign nucleic acid-induced immune response.^90^ DNA nanostructures were shown to elicit a slight dose-dependent immune response up to 10 nM DNA concentration, but with similar immunogenicity in the 10–100 nM range.^89^ Both in vitro and in vivo examination for proinflammatory markers showed a significant elevation in CD69, suggesting activation of immune cells; however, no cytokine storm was detected. Further, the inflammatory markers showed a complete decline before the tenth day. Related to cytotoxic effects from foreign DNA, a majority of the studies indicate that DNA nanostructures do not induce any significant effects on tested cell viability when tested at DNA concentrations covering the 1–500 nM range.^45,56,57,63,90,91^

### Biodistribution

DNA nanostructure shape, dosage, and route of administration influence the in vivo biodistribution and toxicity in mice. Triangular DNA origami structures accumulated in multiple organs when compared to a square lattice rod-shaped origami structure.^89^ Further, intravenous administration resulted in faster clearance than the intraperitoneal route, with only a minor proinflammatory response. Similarly, when comparing the circulation half-lives and biodistribution of DNA tetrahedra and polymer-nucleic acid hybrid nanoparticles, both types of nanostructures showed a short half-life but with slight differences (9.88 and 19.8 min, respectively), indicating that the presence of polymer slowed renal clearance. Both cases showed rapid distribution of DNA nanostructures into tissues, with the highest accumulation recorded in both renal and hepatic tissues.^92^ Such renal accumulation has also been observed for triangular and flat rectangular DNA nanostructures in healthy mice and those with acute kidney injury, followed by slow clearance in urine over a period of 24 h.^93^ This phenomenon is attributed to the compact and negatively charged surface of DNA nanostructures that minimize enzyme and protein interactions with the structure relative to unfolded scaffolds and limit their accumulation in other organs such as the liver and spleen.

### Targeted Delivery

Another important aspect to enhance DNA nanostructure pharmacokinetics is the ability to target certain tissues, thus minimizing off-target side effects. Targeting DNA nanostructures to specific tissues can be achieved by passive strategies, such as size- or shape-dependent accumulation in certain tissues. This strategy can also be combined with site-specific drug release at the target tissue when the nanostructure reconfigures at low pH in tumor^94^ or inflammatory environments to release the drug.^95^ In active targeting, DNA nanostructures are attached to antibodies or aptamers that bind to unique cell receptors overexpressed in certain tissues or diseased areas. The programmable nature of DNA becomes of high utility for this purpose, since both spatial and stoichiometric control of targeting molecules, along with siRNA and other functionalities, can be achieved. This feature allows targeting of cells or tissues that rely heavily on receptor clustering. For instance, the number and spatial arrangement of immune-stimulating adjuvants (CpG) and viral antigens functionalized on DNA origami square block named ‘Dorivac’ had an effect on the immune stimulation, producing a significantly high population of bone marrow-derived dendritic cells at a CpG distance of 3.5 nm.^96,97^

### Pharmacokinetic Techniques for Studying DNA Nanostructures

Conventional pharmacokinetic studies rely on techniques that measure drug concentration in the blood across different time points, yielding parameters such as area under the curve (AUC), terminal half-life (t1/2), effective concentration, therapeutic index, volume of distribution, and clearance.^98,99^ However, there are additional parameters to consider for DNA nanocarriers. DNA nanostructures require further analysis that focuses on their structural integrity, since most of the intended applications are based on the shape and spatial organization of different functionalities. A recent study assessed the structural integrity of a DNA origami nanostructure in vivo by ligation of adjacent staple pairs.^100^ The nanostructures were administered, and the staples were later amplified and quantified using qPCR. Unlike standard fluorophore tracking, this system provided more accurate quantification of intact structures. Although pharmacokinetic parameters were successfully identified for several shapes of DNA nanostructures, including polymeric and lipid coatings, the correlation between design aspects and its effect on siRNA delivery remains unclear. In addition, most DNA nanostructures intended for siRNA delivery carry other functionalities for targeting and cell uptake enhancement. Pharmacokinetic analysis for full constructs along with consequent effects on pharmacokinetic parameters is mandatory to obtain a complete profile of DNA nanostructures.

## Loading of siRNA on DNA Nanocarriers and Release Strategies

siRNAs are attached to DNA nanocarriers by using different strategies. The most common approach is to use single-stranded overhangs on the nanostructure and extend the siRNAs with complementary overhangs for Watson–Crick-Franklin base pairing between the cargo and the carrier (Figure 4a), such as attachment to single-stranded overhangs on a DNA tetrahedron^101–103^ or within DNA origami nanotubes.^43^ Alternatively, one of the strands of the siRNA duplex could be the part of the nanostructure as an extension of one of the component strands, and the other strand is hybridized to make the siRNA cargo (Figure 4b).^104^ In another approach, siRNA cargo can be integrated into the nanostructure framework (Figure 4c). For example, complementary base pairing between the single strand containing siRNAs with ssDNA on a polymer brush led to the cross-linking of the polymer brush and formation of a nanogel.^44^ In this method, siRNA can be loaded by a simple mixing of the siRNA cargo with a DNA-grafted polymer brush. Loading of the siRNA led to an increase in the size of the nanogel from 20 nm to 1.2 μm. Since siRNA performs a structural role in the formation of the DNA nanostructure, the release of the siRNA cargo leads to the disassembly of the nanostructure.

**Figure 4:**
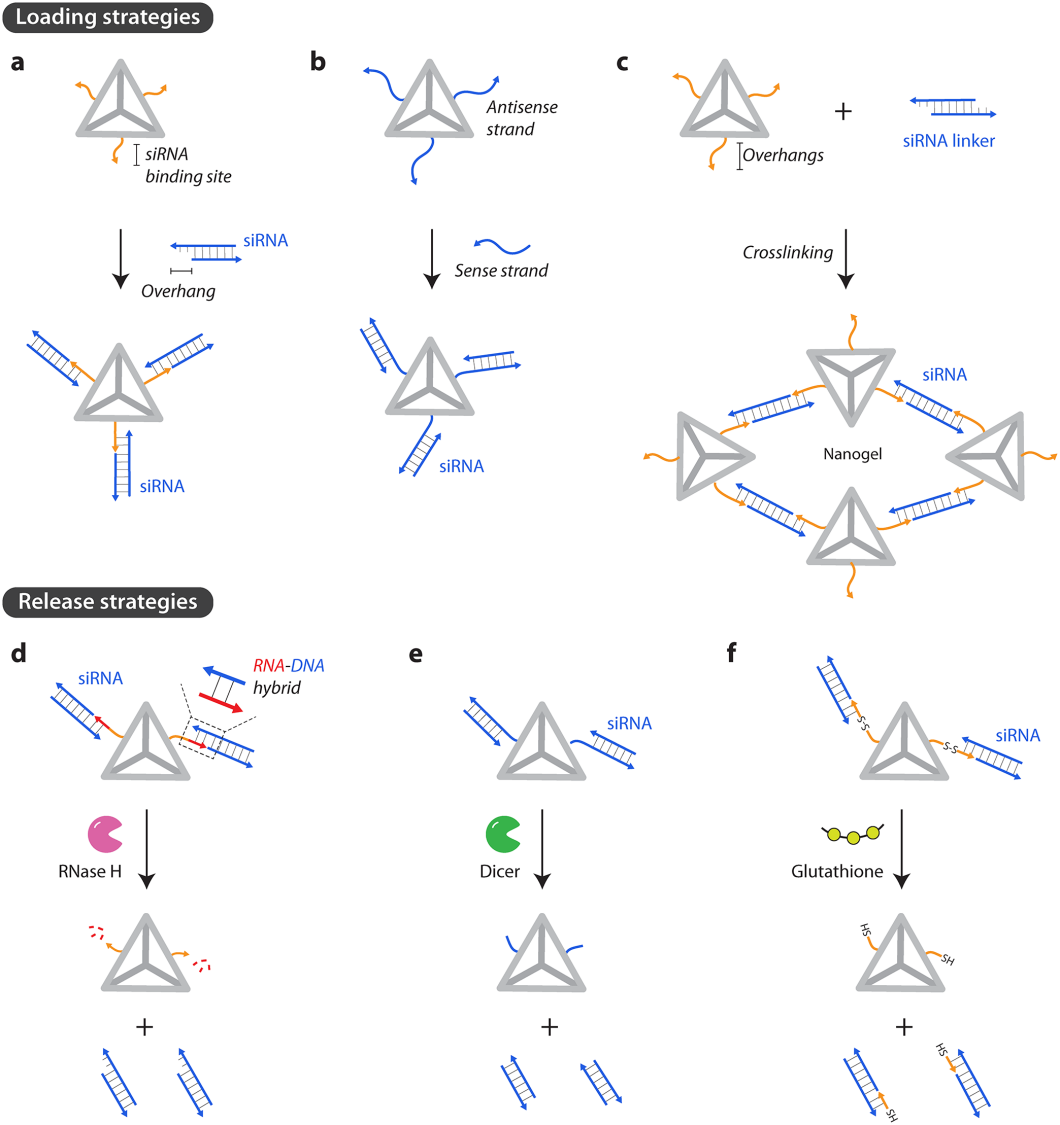
Loading and release of siRNAs. (a) Attachment to single-stranded overhangs on DNA nanostructures. (b) Antisense strand is part of the DNA nanostructure that can bind to the sense strand of the siRNA. (c) siRNA linkers can connect DNA nanostructures to create a nanogel. (d) Release of siRNA by RNase H activity, (e) Dicer activity, and (f) reduction of disulfide linkages by glutathione.

Intracellular delivery of siRNA alone does not guarantee effective gene silencing, and the siRNA must be readily available to bind to Argonaute protein. Successful release of siRNA cargo from the nanostructure is therefore crucial. Two main strategies have been used to achieve this: Responsive siRNA release and strategic spatial placement of siRNA on DNA nanostructures. Examples of stimuli used for triggered release of siRNA in the cytosol of the target cell include enzymatic activity, the reducing environment of the cytoplasm, pH, and strand displacement. By using RNA-DNA hybrids as the linker between siRNA and the nanostructure, RNase H can be used to trigger the cargo release (Figure 4d). For example, siRNAs were released from the nanogel matrix by RNase H, which selectively cleaves the RNA molecules in DNA-RNA hybrids.^44^ Similarly, enzymes such as Dicer^105^ and apurinic/apyrimidinic endonuclease 1 (APE1)^106^ have been used to release siRNAs from DNA nanocarriers (Figure 4e). Dicer-based digestion of the single-strand overhang bound to the polymer-grafted DNA led to the release of the siRNA drugs.^105^ Similarly, APE1, a DNA repair enzyme that is usually present in the nucleus but is translocated to the cytoplasm in an inflammatory state, cleaves the apurinic sites on the nanocarrier.^106^ This exposes the encapsulated siRNAs and triggers its release in lipopolysaccharide-pretreated RAW264.7 cells. Another strategy is to use intracellular stimuli to release the cargo. For example, intracellular reduced glutathione (GSH) can be used to trigger the release of the siRNA and a small-molecule drug from the nanocarrier (Figure 4f).^43^ The reduction of the disulfide linkages in a locking strand by the GSH caused the nanostructure to reconfigure from a tubular state into an open state, exposing the internal cargo. GSH also triggered the reduction of disulfide linkages between siRNA and its overhang, leading to the release of siRNA cargo. Environmental stimuli such as pH changes can also be used to trigger the release of siRNA cargo. For example, siRNAs against regulatory associated protein of mTOR (Raptor) was released from DNA tetrahedron^48^ at low pH when the tetrahedra disassembled due to a structural transformation of the C-rich region of one of the DNA strands into an i-motif. In addition, single-stranded domains within a DNA prism can act as a trigger for mRNA or miRNA to release siRNA mounted inside the prism cavity via toehold-mediated strand displacement.^107^

Spatial organization of siRNAs on the surface of DNA nanostructures is another route to render them accessible for mRNA and RNA silencing proteins. For example, siRNAs attached to the side of 1D hairpin DNA tiles had higher gene silencing effect than those mounted at the center.^108^ In addition, the shape of the DNA nanostructure also dictates the mechanism of gene silencing. siRNA placed on the apex of the 3D tetrahedron showed gene silencing effects at both the mRNA and protein levels, while siRNA attached on 1D nanostructures showed a gene silencing effect only at protein levels, possibly due to the steric hindrance of the 1D nanostructure that prevented the formation of the RISC protein complex responsible for mRNA cleavage.

## DNA Nanocarriers for Therapeutic siRNA Delivery

DNA nanocarriers have been used to deliver siRNAs targeting various diseases. These studies include a range of diseases from humans to animals, with some analysis performed in model cell lines and others performed in animal models (Table 1).

**Table 1:** Delivery of siRNA Using DNA Nanocarriers for Different Diseases^a^

disease	model organism	nanocarrier	siRNA/small-molecule drug	response	administration	references
Breast cancer	MDA-MB-231 tumor-bearing mice	DNA nanogel	Anti-PLK1	Reduction in Ki67-positive tumor cells	I.V.	^44^
	Tumor-bearing humanized nude mice	DNA nanowire	Anti-PLK1 and doxorubicin	Inhibition of tumor growth	I.V.	^109^
	MCF-7R tumor-bearing mice	DNA origami	Anti-Bcl2, anti-P-gp and doxorubicin	Reduction of tumor growth	I.V.	^43^
Non-small-cell lung cancer	Immunodeficient mouse model	DNA nanotube	Anti-KRAS^G12C^ and metformin	Reduction of tumor growth	I.V.	^52^
Glioblastoma	CB17 SCID mice	Spherical nucleic acids	Bcl2L12	Impaired tumor growth	I.C.	^110^
Aging	Chemotherapy-induced senescent mice model	DNA tetrahedra	Anti-Raptor	Increase in the mean life span	I.P.	^48^
Acute lung injury	Acute lung injury mice model	DNA tetrahedra	Anti-mTOR and spermidine	Shift in the macrophage polarization	I.V.	^113^
Acute kidney injury	BALB/c mice	l-DNA tetrahedra	Anti-p53	p53 knockdown	I.V.	^49^
	C57BL/6 mice	DNA tetrahedra			I.V.	^114^
Hypercholesterolemia	BALB/c mice	DNA tetrahedra	Anti-ApoB1	Reduction in the lipid levels in the blood	I.P.	^104^
Inflammation	C57BL/6	DNA tetrahedra	Anti-TNFα	Reduction in anti-TNFα mRNA level	I.P.	^42^
Ulcerative colitis	C57BL/6 J	DNA nanotube	Anti-TNFα and anti-integrin α4	reduced intestinal macrophage recruitment and T-cell homing	I.V.	^106^

a Abbreviations: PLK1, Polo-like kinase 1; Bcl2, B-cell lymphoma 2; P-gp, P-glycoprotein; KRAS, Kirsten rat sarcoma viral oncogene; Raptor, Regulatory associated protein of mTOR complex 1; mTOR, Mechanistic target of rapamycin; ApoB1, Apolipoprotein B; TNFα, Tumor necrosis factor alpha; I.V., intravenous; I.P., intraperitoneal, I.C., intracranial.

### Cancer

A DNA nanogel was used to deliver siRNA targeting polo-like kinase 1 (PLK1), which is overexpressed in human breast cancer cell line (MDA-MB-231).^44^ siRNA knockdown of PLK1 by the nanogel led to an apoptosis rate comparable to that of lipofectamine-transfected siRNA. Intravenous administration of the nanogel carrying anti-PLK1 siRNA at a dose of 1 mg/kg per injection into MDA-MB-231 tumor-bearing mice led to a higher PLK1 knockdown than the lipofectamine control. Further, siRNA-nanogel reduced the proliferation of Ki67-positive tumor cells.

Since cancer is a complex disease, a combinatorial therapeutic approach with drugs that act by various mechanisms is often preferred. The multifunctionality of DNA nanostructures allows the delivery of several types of drugs for combinatorial therapy. For example, a spherical DNA nanostructure was created from multiple DNA tetrahedra assembled on a long DNA nanowire, and used to load anti-PLK1 siRNA and a DNA-intercalating drug doxorubicin.^109^ Interactions between the palindromic sequences of the nanowire lead to the formation of spherical nanostructures. The nanostructures were functionalized with AS1411 aptamer to specifically target nucleolin proteins, which are overexpressed in cancer cells. Treatment with doxorubicin/siRNA-loaded DNA nanostructures resulted in a 40% knockdown of PLK1 mRNA levels in HeLa cells and led to the inhibition of tumor growth in tumor-bearing humanized nude mice. In another study, tubular DNA origami was used to deliver siRNA and doxorubicin simultaneously in vitro and in vivo.^43^ In this case, the siRNAs encapsulated within the DNA origami carrier were protected from nuclease degradation. Cellular uptake of the origami structure was promoted by integration of the cell-penetrating peptide, trans-activator of transcription (TAT) peptide, into the DNA origami nanocarrier. Treatment of a combination of Bcl2 and P-gp targeting siRNAs and doxorubicin to the human breast adenocarcinoma (MCF-7R) cell line using the DNA nanocarrier showed reduced expression of Bcl2 and P-gp genes. Intravenous injection of the nanocarrier with the siRNA and doxorubicin cargo into mice displaying MCF-7R tumors led to reduced tumor growth. Similarly, DNA nanotubes that deliver small-molecule drug metformin and siRNA exhibited a synergistic antitumor effect against KRAS-mutated non-small-cell lung cancer (NSCLC) in vitro and in a mouse model.^52^ KRAS controls cell growth and cell death, and its mutation leads to tumorigenesis and tumor maintenance. Treatment of H358 cells with nanotubes carrying metformin and siRNA against KRAS with the G12C mutation led to a 41% decrease in cell viability. Systemic administration of the DNA nanotube carrying the combination of metformin and siRNA into the H358 cell-implanted immunodeficient mouse model led to a reduction in KRAS mRNA levels in tumors while not affecting other organs such as the lungs and kidneys. The combination of the two drugs showed better therapeutic value compared with either metformin or siRNA.

Spherical nucleic acids (SNAs) have been used to deliver siRNAs to intracranial tumor.^110^ SNAs composed of a gold nanoparticle core with their surface functionalized with siRNAs targeting glioblastoma oncogene Bcl2Like12 (Bcl2L12) showed reduced Bcl2L12 protein expression as well as increased effector caspase-3 and caspase-7 activation in patient-derived glioma-initiating cells. Upon systemic administration, SNAs produced Bcl2L12 knockdown in patient-derived xenograft-bearing mice following accumulation in the extravascular tumor parenchyma. A first-in-human phase 0 clinical trial of siBcl2L12-SNA showed that the nanostructure is safe when injected intravenously at a dose of 0.04 mg/kg.^111^

### Aging

Raptor is a key regulator of the aging process and is a component of mTORC1. Delivery of Raptor-targeting siRNA using DNA tetrahedra delayed aging by effectively inhibiting mTORC1 signaling.^48^ siRNA-loaded tetrahedra reduced the expression of senescence markers (p21 and p16) and senescence-associated secretory phenotype markers (IL6 and tumor necrosis factor α (TNF-α)) along with a reduction in the level of reactive oxygen species in senescent fibroblasts. Knockdown of Raptor inhibits senescence-associated β-galactosidase (SA-β-Gal) activity, which reduces the level of expression of p16^INK4a^ and attenuates cellular senescence. Administration of siRNA-loaded tetrahedra to chemotherapy-induced senescent mice model showed an increase in the mean life span by 53.8% and reduced the p21^CIS1^ positive cells in the liver, indicating that it delayed aging. Further, the use of the tetrahedra to deliver siRNA to naturally aged mice showed indications of improved motor function, endurance, and spontaneous exploration.

### Acute Lung Injury

Macrophage polarization is involved in the development of acute lung injury.^112^ Since DNA nanostructures are readily taken up by macrophages, they are well suited for the delivery of siRNA drugs that alter macrophage polarization involved in the development of acute lung injury.^113^ Tetrahedral DNA nanostructures loaded with mTOR-targeting siRNA and spermidine reduced the expression of mTOR by 80% and induced M2 macrophage polarization, with spermidine and anti-mTOR siRNA acting synergistically. Its administration into acute lung injury mice model showed a 60% reduction in the mTOR expression in the lungs and promoted the phenotype transformation of macrophages, indicating a shift in the macrophage polarization while producing an anti-inflammatory effect.

### Acute Kidney Injury

Tetrahedra assembled from DNA strands containing different sugar modifications such as l-deoxyribose, 2′O-Me ribose, and 2′ fluoro ribose are used as nanocarriers for delivering siRNAs to the kidneys.^49^ siRNA targeting P53 were loaded on to the l-DNA tetrahedra by an overhang on the sense strand and delivered to TCMK1 cells, reducing mRNA levels by 60%. The modifications enhanced the serum stability of the structures by 50%. Intravenous administration of siRNA-loaded l-DNA tetrahedra led to a 70% decrease in the p53 mRNA levels in the kidney of BALB/c mice. In addition, DNA tetrahedra containing three cholesterol modifications at the vertices have also been used as siRNA carriers for acute kidney injury.^114^ The siRNA targeting p53 was attached to the fourth vertex of the tetrahedra, inducing the knockdown of p53 in both cell lines and animal models. Cholesterol conjugation improved the cellular uptake properties of the tetrahedra in the TCMK1 primary cell line. In animal model studies,^114^ the nanocarriers were absorbed by the proximal and distal tubules of the kidney in the C57BL/6 mice upon tail vein injection. Further, accumulation of the nanocarrier in the kidney did not produce any adverse effects, and the levels of the renal damage biomarkers were unaltered.

### Hypercholesterolemia

Hypercholesterolemia is a disease characterized by high levels of cholesterol in the blood and is linked to mutations in ApoB1 gene.^104^ Knockdown of ApoB1 in the liver is an effective therapeutic strategy for hypercholesterolemia. As tetrahedral DNA nanostructures have been shown to accumulate in the liver,^115,116^ they are effective nanocarriers for targeted delivery of anti-ApoB1 siRNA to the liver.^104^ On treatment with siRNA-carrying tetrahedra, HepG2 cells showed a 40% decrease in the expression of ApoB1 mRNA. Intraperitoneal injection of the nanostructure carrying siRNAs into BALB/c mice led to their accumulation in the liver after 2 h and a reduction in the level of ApoB mRNA by 50% in the liver lysate. Successful knockdown of ApoB was further confirmed by a reduction in lipid levels in the blood by 20–30%.

### Inflammation

TNFα targeting siRNA was embedded within a tetrahedral DNA nanostructure by overhangs on either side of the antisense strand.^42^ One of the component strands of the tetrahedra was C-rich and could transform into an i-motif at low pH. This structural reconfiguration disassembles the tetrahedron and releases the cargo siRNA under acidic conditions, similar to the environment in lysosomes. Treatment with the siRNA-loaded nanocarrier led to downregulation of TNFα in macrophages by 75%. In vivo studies in a mouse model showed a reduction in the mRNA level by 50% following intraperitoneal injection.

### Ulcerative Colitis

Ulcerative colitis is a chronic inflammatory condition of the colon and rectum with multiple pathogenic genes and causes. Inhibition of proinflammatory cytokines such as TNFα in colonic macrophages and prevention of T-cell homing at the inflammatory sites are part of the current therapeutic strategies to treat ulcerative colitis.^117^ This can be achieved by a combination therapy of siRNAs that target TNFα and integrin α4. DNA origami nanotubes carrying anti-TNFα and anti-integrin α4 siRNAs accumulated in the intestines of mice with colitis and were internalized by the inflamed cells.^106^ While the DNA nanotube showed an ROS-scavenging property and protected the tissues from oxidative stress, the delivered siRNAs silenced TNFα and integrin α4, resulting in reduced intestinal macrophage recruitment and T-cell homing. Use of DNA nanotubes as a delivery vehicle enabled the selective delivery of the multiple siRNAs in inflamed cells and inhibition of inflammation.

### Swine Fever

Classical swine fever virus (CSFV) causes a highly infectious swine fever in pigs. Therapeutic strategies to reduce viral replication can help mitigate the severe losses to the livestock industry caused by the disease. Simultaneous delivery of siRNAs against CSFV genes C3 and C6 to CSFV-infected PK15 cells using DNA tetrahedra nanocarriers decreased the virus titers by inhibiting the viral replication in the host cells.^118^

## Outlook

The programmability of DNA nanostructures allows the construction of different nanoscale shapes with additional functionalities such as targeting, enhanced biostability, and triggered release. One of the major advantages of using DNA nanostructures as carriers of siRNA is that it allows the loading of multiple types of cargos at the same time, allowing combinatorial therapy. Although multiple siRNAs have been loaded onto a single nanostructure,^106^ controlled delivery of distinct siRNAs at defined individual doses, enabled by precise stoichiometric loading of siRNA cargo, remains to be demonstrated. Recent developments in DNA nanostructure assembly strategies have further improved the efficiency of DNA nanostructures for use in siRNA delivery. The choice of counterions used for nanostructure assembly plays a critical role in determining the efficiency of cellular uptake. Tetrahedral DNA nanostructures synthesized in the presence of spermidine and loaded with mTOR-targeting siRNA were internalized into bone marrow-derived macrophages ∼1.5 times more than those synthesized using Mg^2+^ as the counterion.^113^ Thus, the ability to assemble DNA nanostructures in different counterions other than the typically used magnesium has allowed the use of DNA nanostructures in different settings. Another factor that affects cellular uptake is protein corona composition, and there are only a limited number of studies that explore how to modulate DNA nanostructures to better control protein corona formation.^64,65,81^ Some of these strategies include coating with polymers such as PEG, and the shielding effect becomes more effective with increasing PEG chain length or grafting density.^67^ However, increasing these two features may affect the cellular uptake since PEG also shields DNA nanostructures from cell membrane receptor interaction, a phenomenon referred to as the “pegylation dilemma”.^119^ Such polymer coatings also enhance the biostability and biodistribution of DNA nanostructures, but may not be preferred as the accumulation of these polymer-coated DNA nanostructures within tissues for extended time periods may lead to potential toxicity.^120^ In addition, PEG was reported to induce antibody-mediated immune reactions ranging from minor inflammation to severe immunological effects^121^ or rapid clearance as in the case of mRNA vaccines.^122^ Another key parameter, endosomal escape, while still a challenge, is being addressed by modifications to the DNA nanostructures or the use of additives.^10^ The capability of using peptides to achieve endosomal escape is another strategy that can be applied to DNA nanocarriers.^123^

Clinical translation of DNA nanocarrier-mediated siRNA delivery faces major challenges, including those related to nanostructure manufacturing processes, pharmacokinetic and pharmacodynamic properties of DNA nanostructures, and regulatory aspects of this novel therapeutic modality. For DNA nanostructures to be translated into real-life siRNA delivery carriers, more systematic investigations would aid in better understanding of the delivery efficiency as well as to create metrics for nanostructure design and assembly.^70,124–128^ Given the large diversity of DNA nanostructures, developing good manufacturing practices (GMP), robust quality control methods, stability assays to verify the nanostructure assembly, and achieving batch reproducibility are crucial. Large-scale synthesis of some nanostructures has been achieved^129^ but has not been shown for many, including DNA tetrahedra, which is commonly used for these applications. Lack of high-throughput methods for analysis of fine structural features and the overall morphology of large nanostructures is a major bottleneck. However, routine chromatography and spectroscopic methods can be easily adapted for smaller DNA nanostructures such as tetrahedra.^130,131^ Controlled spatial organization and stoichiometry of siRNA cargo on multivalent DNA nanocarriers may require specialized assays. The pharmacokinetics of nanocarriers is likely to be influenced by the nucleases. Since the immune recognition of the intact nanostructure could be different from that of the nuclease degradation products, the predictability of the pharmacokinetics of these materials is challenging. This reinforces the need for biostable nanostructures for drug-delivery applications. Further, the immunogenic response may need to be tested for each shape and size of DNA nanostructures. Regulatory frameworks for DNA nanostructure-based delivery platforms might align with those established for oligonucleotide therapeutics and nanomaterials.

Although lipid nanoparticles (LNPs) have emerged as effective vehicles for the delivery of nucleic acid drugs over the past few years, the full therapeutic potential of the next-generation nucleic acid drugs, such as ASOs, siRNAs, and aptamers, requires more sophisticated nanocarriers. As a nanomaterial, DNA offers a dynamic and programmable scaffold for constructing delivery vehicles with precise control over cargo-loading, the ability to carry multiple drugs, and the release of drugs at the site of action prompted by site-specific stimuli. Compared to LNPs, small nanostructures, such as DNA tetrahedra, exhibit lower loading capacity. However, DNA origami strategies allow for the construction of nanocapsules with desired encapsulation volume and defined drug-loading capacity and distribution within the carrier.^132^ Presently, a direct quantitative comparison between LNPs and DNA nanocarriers is complicated by differences in formulation, design, dosing, and disease models. Nevertheless, DNA-based platforms are increasingly recognized as customizable alternatives for lipid-based carriers.

Overall, DNA nanostructures have been shown to be efficient siRNA delivery carriers, with progress in the analysis of therapeutic efficiency in vitro, in cells, as well as in animal models. Akin to the crew navigating the bloodstream in the movie The Fantastic Voyage, DNA nanostructures can one day function as programmable devices to directly deliver drug payloads into the body.
